# DAART: a deep learning platform for deeply accelerated adaptive radiation therapy for lung cancer

**DOI:** 10.3389/fonc.2023.1201679

**Published:** 2023-07-06

**Authors:** Hamed Hooshangnejad, Quan Chen, Xue Feng, Rui Zhang, Reza Farjam, Khinh Ranh Voong, Russell K. Hales, Yong Du, Xun Jia, Kai Ding

**Affiliations:** ^1^Department of Biomedical Engineering, Johns Hopkins School of Medicine, Baltimore, MD, United States; ^2^Department of Radiation Oncology and Molecular Radiation Sciences, Johns Hopkins School of Medicine, Baltimore, MD, United States; ^3^Carnegie Center of Surgical Innovation, Johns Hopkins School of Medicine, Baltimore, MD, United States; ^4^Department of Radiation Oncology, City of Hope Comprehensive Cancer Center, Duarte, CA, United States; ^5^Carina Medical, Lexington, KY, United States; ^6^Division of Computational Health Sciences, Department of Surgery, University of Minnesota, Minneapolis, MN, United States; ^7^Department of Radiology and Radiological Science, Johns Hopkins School of Medicine, Baltimore, MD, United States

**Keywords:** deep learning, machine learning, image synthesis, adaptive radiation therapy (ART), artificial intelligence, non small cell lung cancer (NSCLC)

## Abstract

**Purpose:**

The study aimed to implement a novel, deeply accelerated adaptive radiation therapy (DAART) approach for lung cancer radiotherapy (RT). Lung cancer is the most common cause of cancer-related death, and RT is the preferred medically inoperable treatment for early stage non-small cell lung cancer (NSCLC). In the current lengthy workflow, it takes a median of four weeks from diagnosis to RT treatment, which can result in complete restaging and loss of local control with delay. We implemented the DAART approach, featuring a novel deepPERFECT system, to address unwanted delays between diagnosis and treatment initiation.

**Materials and methods:**

We developed a deepPERFECT to adapt the initial diagnostic imaging to the treatment setup to allow initial RT planning and verification. We used data from 15 patients with NSCLC treated with RT to train the model and test its performance. We conducted a virtual clinical trial to evaluate the treatment quality of the proposed DAART for lung cancer radiotherapy.

**Results:**

We found that deepPERFECT predicts planning CT with a mean high-intensity fidelity of 83 and 14 HU for the body and lungs, respectively. The shape of the body and lungs on the synthesized CT was highly conformal, with a dice similarity coefficient (DSC) of 0.91, 0.97, and Hausdorff distance (HD) of 7.9 mm, and 4.9 mm, respectively, compared with the planning CT scan. The tumor showed less conformality, which warrants acquisition of treatment Day1 CT and online adaptive RT. An initial plan was designed on synthesized CT and then adapted to treatment Day1 CT using the adapt to position (ATP) and adapt to shape (ATS) method. Non-inferior plan quality was achieved by the ATP scenario, while all ATS-adapted plans showed good plan quality.

**Conclusion:**

DAART reduces the common online ART (ART) treatment course by at least two weeks, resulting in a 50% shorter time to treatment to lower the chance of restaging and loss of local control.

## 1 Introduction

It is the most common cause of cancer-related death (1). Multiple studies have reported that lung cancer with a higher mortality rate is associated with treatment delay (2–5). Clinical studies have found that 13% of patients may develop new lymph node involvement, site of disease, and a chance of increase in the stage at 4 weeks (6), and potentially, a 14% loss of local control per week with prolongation of the treatment course (7). More recent studies have suggested an association between prolonged time to treatment initiation (TTI) and mortality for pancreatic and lung non-small cell lung cancer NSCLC, where each week of increase in TTI was associated with 3.2% and 1.6% mortality increases in stages I and II (4). In 2015, Samson et al. reported that delay in resection could be associated with upstaging and decreasing median survival, and for each week of delay to surgery, mortality can increase by 0.4% (3). A recent study reported patients with TTI<45 days had a median overall survival of 70.2 months, while patients with TTI >45 days had a median 61.5 overall survival (5).

Radiation therapy (RT) is a highly effective and preferred treatment for medically inoperable NSCLC (8). NSCLC accounts for 87% of lung cancer diagnoses (9), and over 60% of patients with NSCLC require radiotherapy at least once during the course of their disease (10, 11). However, the current RT workflow consists of numerous steps that result in a considerable delay before treatment initiation. As mentioned, the delay in NSCLC treatment initiation may cause complete restaging (6) and thus, it can be very beneficial for patients to expedite TTI after diagnosis. Therefore, the question arises as to how the current workflow can be optimized.

**Figure 1:**
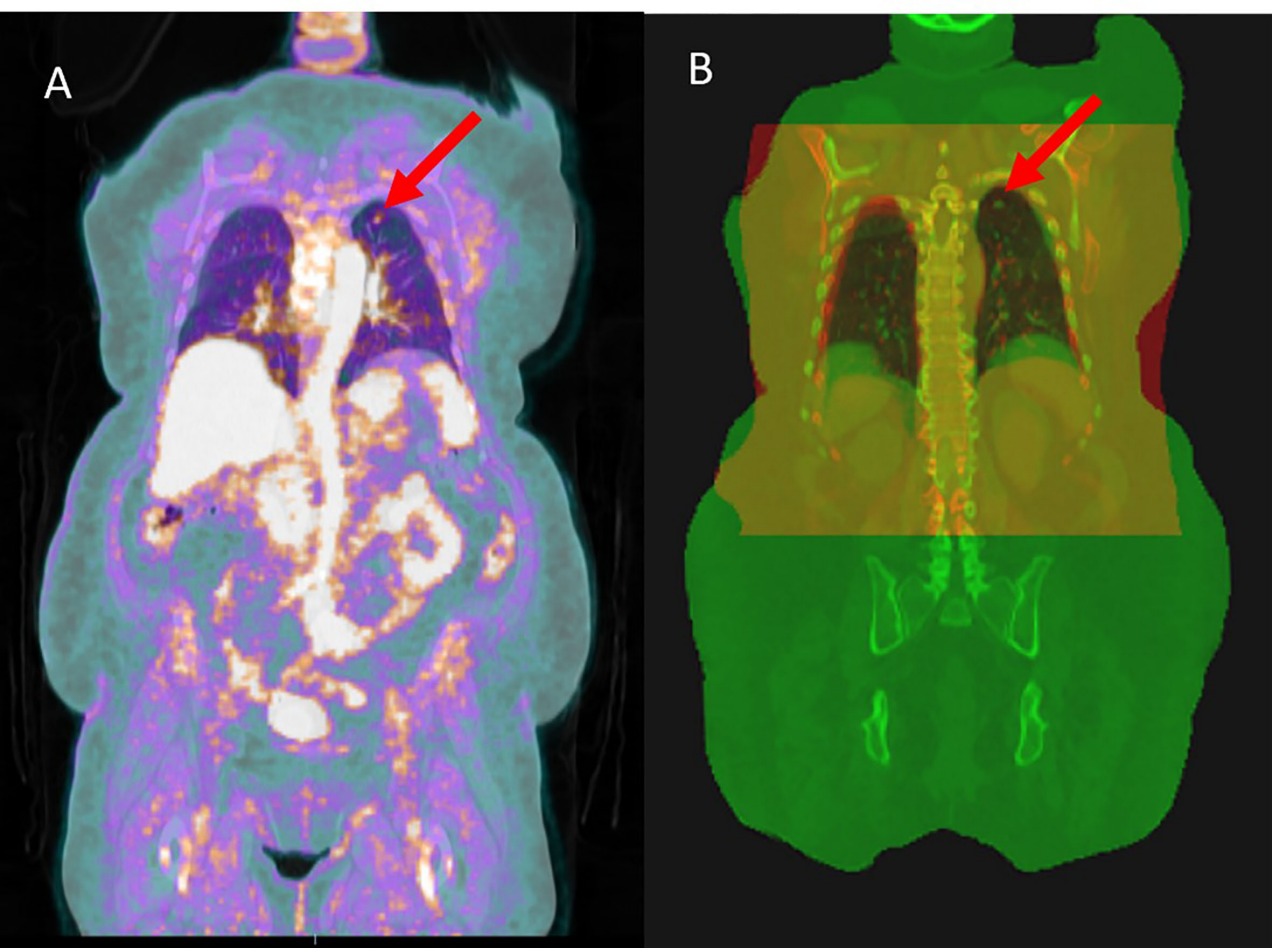
**(A)** PET scan overlaid on PET/CT scan, used for the initial diagnosis of the tumor (red arrow). The tumor is recognized by its high uptake of fluorine-18-fluorodeoxyglucose (F-FDG) **(B)** pCT scan (red) overlaid on the PET/CT scan (green), and the overlapping area is shown as yellow. One of the main differences between the two scans is the diaphragm level owing to the motion management technique.

The current workflow is considerably lengthy owing to the many sources of delay (12–18), which imposes a huge burden on patients and their caregivers (19, 20). A major source of delay is the need for several appointments and separate image acquisitions, such as the acquisition of a diagnostic positron emission/computed tomography (PET/CT) scan (**Figure 1A**) in the radiology department and a simulation/planning scan (pCT) in the radiation oncology department. It causes a median delay of 15 days for patient diagnosis and an additional 15 days from diagnosis to treatment initiation (21). A 2015 study also reported a median duration of 27 days from diagnosis to treatment initiation (16). A 2020 study reported an increase in the median TTI from 35 to 39 days between 2004 and 2013 (5). Considering the chance of complete restaging with a delay of four weeks (6) we addressed the median 15 days delay between diagnosis and treatment initiation.

**Figure 2:**
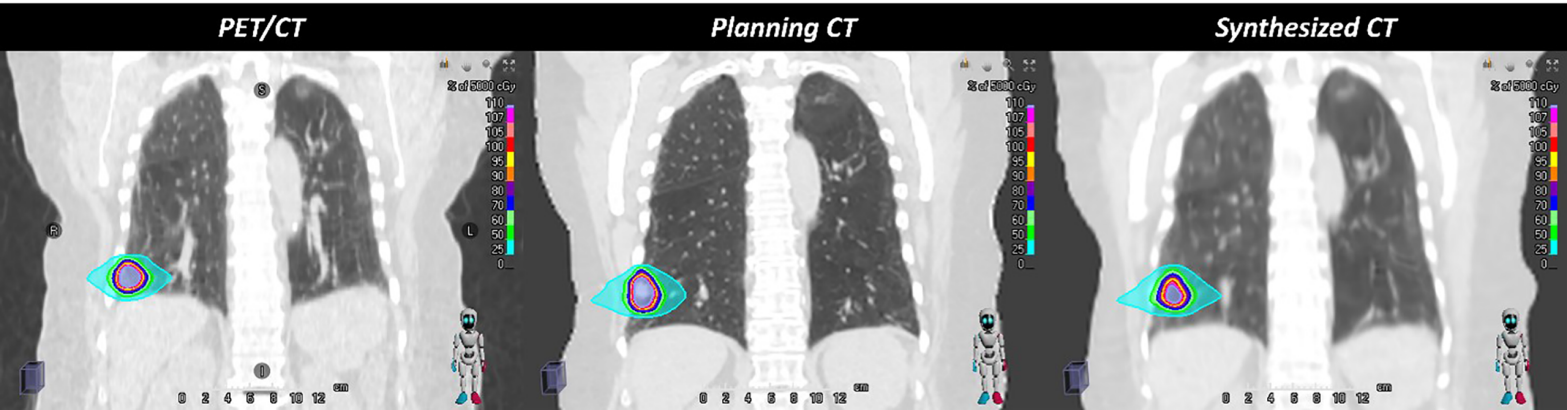
The figure demonstrates the anatomical differences due to the breath-hold motion management technique, which causes a large dosimetric difference between the RT plans recalculated on planning CT and PET/CT scans. However, the deepPERFECT synthesized CT scans captured the anatomical changes and the dose difference between the planning CT and synthesized CT was significantly reduced. Therefore, PET/CT cannot be directly used in treatment planning.

In addition to being used for target delineation, PET/CT has been shown to be compatible with RT planning (20, 22) and is used for RT treatment of prostate and head and neck cancer (23–25). However, in practice, however, the acquisition of planning CT (pCT) is still required for lung cancer RT. This is because of the different image acquisition settings and motion management techniques used for acquiring PET/CT and pCT images. The PET/CT scan is performed in the radiology department using a curved couch top for patient comfort, whereas pCT is acquired in radiation oncology and uses a flat couch top, for patient position reproducibility. Moreover, to minimize tumor motion, the widely used active breath-hold coordinator (ABC) or similar techniques are used during pCT acquisition and RT treatment initiation. As a result, there is a notable difference between the patient body shape and diaphragm level on the two scans (**Figures 1B**, **2**), which results in a large dosimetric difference between RT plans designed on planning CT and PET/CT (**Figure 2**). This is the reason why PET/CT cannot be used directly for treatment planning. Although a PET/CT dedicated simulator approach is also available, the cost of staffing, purchasing, and maintaining such a device can be prohibitive (26) and no clear clinical difference has been observed (27).

The advent of current advances in the application of artificial intelligence (AI) (28, 29), graphical processing unit (GPU)-based dose calculation engines (30–32), and automatic and semi-automatic segmentation and planning systems (33) have made the many steps of the online adaptive workflow to be performed faster and in the online ART timeframe (34), such as the recently developed ART systems such as ViewRay’s MRIdian A3i (ViewRay, Oakwood Village, Ohio, USA) and Varian Ethos system (Varian Medical Systems, Palo Alto, CA). For instance, the Ethos system can perform online ART in less than 20 min (35–37). Although these advanced ART systems increase the accuracy of dose delivery and shorten the same-day treatment course, they still do not help reduce the long delay between diagnosis and treatment initiation, as pCT is still required for initial RT planning.

**Figure 3:**
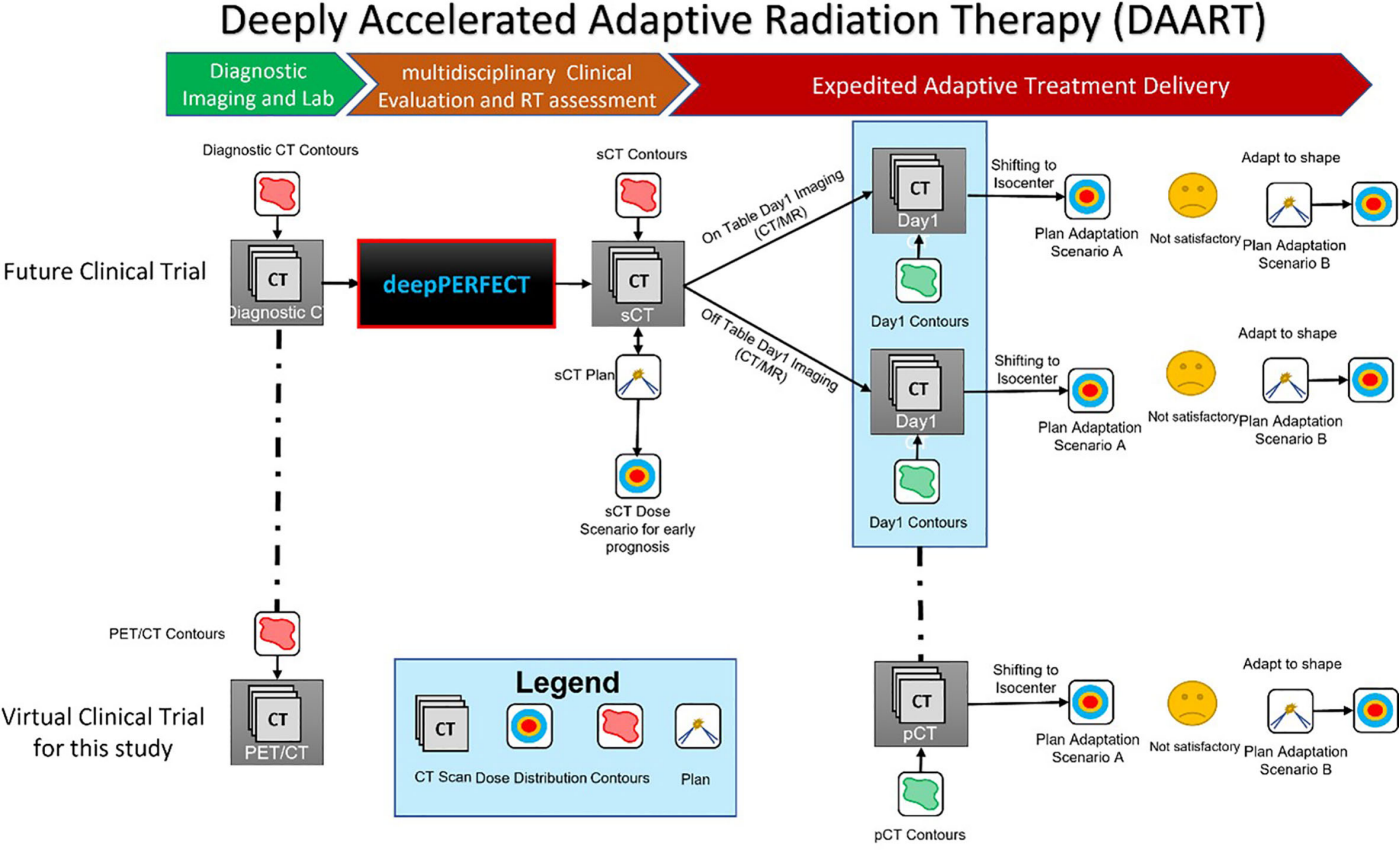
Illustration of the deepPERFECT-enabled deeply accelerated adaptive radiation therapy (DAART) workflow. The top pathway shows the implementation of deepPERFECT in future clinical trials, where the generated sCT from diagnostic PET/CT enables the design of a preliminary plan (sCT plan). Two-step off-table and on-table plan adaptation (first, ATP and then ATS) adapts the sCT plan to planning CT (pCT). The bottom pathway shows the virtual clinical trial in this study, where off-table adaptive planning was used for the two-step adaptation.

Thus, we have devised a new expeditious RT workflow by developing a novel deep learning method for Planning External-beam Radiotherapy Free from Explicit simCT or deepPERFECT to address the considerably long wait time between NSCLC diagnosis and RT treatment initiation (**Figure 3**). Using deepPERFECT, we synthesized the treatment Day1 pCT or sCT from a diagnostic PET/CT scan, previously applied to pancreatic cancer RT (20, 38–53). The sCT scan is used to design the initial RT treatment plan (sCT plan) for early assessment and evaluation of the RT plan and to decide on dose trade-offs. Therefore, the design and verification of the initial RT plan are no longer delayed owing to the lack of pCT scans. On treatment Day1, the sCT plan was adapted to the treatment delivery patient setup using on/off table imaging for same-day online ART such as on-table adaptive therapy methods such as Ethos or same-day off-table online ART, using Raystation adaptive treatment planning (RaySearch Laboratories, Sweden). Therefore, deepPERFECT enables deeply accelerated ART or DAART that reduces the current median 4 weeks workflow to 2 weeks from diagnosis to treatment initiation. Additionally, the generation of planning CT from diagnostic CT reduces the patient’s exposure to radiation from multiple CT acquisitions for planning following the ALARA principle as suggested by AAPM TG 75 and 180 (54, 55).

## 2 Materials and methods

### 2.1 Overview

As mentioned above, **Figure 3** shows the proposed DAART approach. This study aimed to demonstrate the practicality of our method by conducting a virtual clinical trial. We used a deepPERFECT-adapted diagnostic PET/CT scan to design the initial treatment plan. Next, we used RayStation off-table adaptive RT planning to adapt the initial sCT scan to pCT. Here, the pCT or planning/simulation CT is equivalent to the treatment Day1 CT in our DAART approach. The adaptation is performed in two steps: first, the adaption to the isocenter, similar to adaptation to position (ATP), and if the plan is not satisfactory (for instance, the 95% target coverage is not acquired), the initial plan is adapted using the adapt-to-shape (ATS) technique. In the ATP scenario, we only shifted the isocenter of the beams and recalculated the dose on the new CT (pCT) without re-optimization. However, for the ATS scenario, first, the iso-center of the beams is shifted; then, using the same (sCT) plan parameters, we reoptimized the plan on the new CT (pCT) and used the new contours.

### 2.2 Data preprocessing pipeline

In this study, we used retrospective data from 15 patients with early stage NSCLC with internal review board (IRB) approval. All patients were treated with standard-of-care stereotactic body radiation therapy (SBRT) at our institution. PET/CT scan and planning CT scan (pCT) were acquired as part of their standard of care for each patient. The scans were acquired with 120 KVp, 200 mA, and 50 cm field of view. Owing to the varying physical dimensions of the scans and GPU limitations, all scans were first resampled to a 2.5 mm voxel dimension. We developed a robust couch removal algorithm to remove the couch prior to registration. Because the treatment couch was identical, it was digitally added to the synthesized CT for accurate dose calculation. Certified physicians delineated all contours.

To obtain the ground truth for training and validation, we aligned PET/CT and pCT images via deformable registration. Specifically, we first performed a rigid registration between CT and PET/CT, that is, diagnosis CT (dCT) and pCT. Since, the dCT is first acquired in the usual workflow, we registered the pCT to the dCT so that the dCT physical dimensions served as the reference dimensions. Next, using an in-house spine segmentation algorithm, we performed a spine-restricted rigid registration between the dCT and resampled pCT. Finally, the dCT was deformably registered to the pCT with spine rigidity penalty. All registrations were performed using the Elastix image-registration algorithm (56–70). Out of the 15 cases, we used 10 for training and five cases for testing and planning. The data were augmented for training by creating random 128 cubic patches, −20 to 20 mm shifts, and −10 to 10 degrees rotations along the superior–inferior axis.

### 2.3 Deep learning model

**Figure 4:**
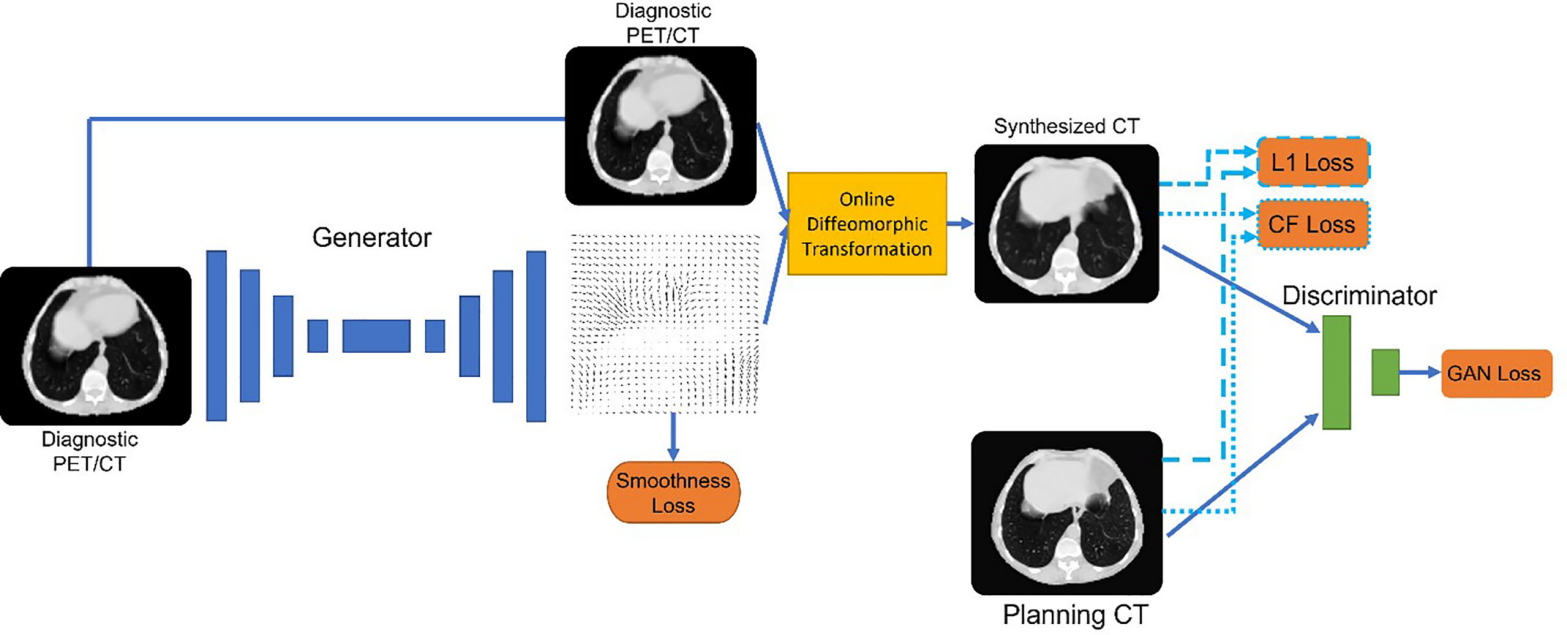
Architecture of deepPERFECT. We illustrated the generator and discriminator pair, along with an online diffeomorphic deformation block, to transform the generated fields into diffeomorphic DVFs. The network was trained using multiple cost functions, including a novel contrast-fidelity term.

A 3D Pix2Pix generative adversarial network (GAN) was used for this purpose (**Figure 4**), which is a general neural network for image-to-image transition, consisting of a generator and discriminator (71). The generator–discriminator pair was simultaneously trained. We used a U-net architecture for the generator, with seven encoding and decoding levels. Each layer consisted of 3D convolutional kernels, batch normalization, and nonlinear activation units. The input to the model single-channel 128 cubic patches of the CT scan and the network generated the corresponding three-channel deformation vector fields (DVFs) for the deformation of dCT to pCT. The discriminator is a convolutional encoder. If the input is a real pair, it should be classified as real, and if it is fake, it should be classified as fake.

The generator was trained to minimize the following loss function:


Eq. 1 $$Generator\;Loss=Adversial\;Loss+\;\lambda_{1}\times Image\;Similarity\;loss+\;\lambda_{2}\times Constrast\;Fidelity\;loss+\;\lambda_{3}\times smoothness\;regulatory\;term$$

As mentioned, the generator was designed to generate DVFs. We chose to generate DVFs rather than CT to preserve CT intensity calibration through careful quality assurance so that they can be used for treatment delivery and dose calculation. Thus, to avoid introducing any inaccuracy in the CT Hounsfield Unit (HU), the model generates DVFs rather than the CT scan. Using the diffeomorphic transformation method, we converted the generated DVFs to diffeomorphic deformation on demand. Consequently, diffeomorphic flows are applied to the image rather than directly to the output of the network. Diffeomorphism guarantees topology preservation and prevents contours from flipping and tearing (72–75). Diffeomorphic DVFs were applied to the input image immediately as part of the training process to obtain the deformed image. Thus, the image similarity loss is:


Eq. 2 image similarity loss=L1 loss (Input, Output)

The next loss term was the novel contrast fidelity term. The contrast fidelity term imposes a heavier weight on the similarity of the features visible in specific window/level image displays to enhance the corresponding features. For instance, for lung CT, the common (window/level) values are (1,600/−600) which enhances lung features such as the pulmonary vessels. We implemented this window/level function by using the following differentiable function:


Eq. 3 $$I_{out}=\left(\frac{1}{1+\exp(-\frac{\left(I_{in}-a\right)}{b}}\right)\times c$$

Here, we used a, b, and c values of 500, 500, and 3,000, respectively to mimic the lung window. Depending on the corresponding sites, a, b, and c can vary to provide a similar display of the image as physicians use in practice. The contrast fidelity loss is then defined as:


Eq. 4 contrast fidelity loss=L1 loss (Soft Contrast(Input), Soft Contrast(Output))

where the Soft Contrast function is defined in Eq. 3. Finally, to enforce smooth deformation fields we used the second-order curvature regulatory term (76) given by


Eq. 5 $$smoothness\;regulatory\;term=\;\int \sum_{j=1}^{3}\|\Delta DVF_{j}{\left(x\right)\|}^{2}dx$$

### 2.4 Radiation therapy planning

The five test cases were planned with volumetric modulated arc therapy (VMAT) SBRT (50 Gy in five fractions) according to our institution’s planning protocol for the lung. To create the planning target volume (PTV), the internal gross target volume (IGTV) by 3 mm. Each patient was asked to hold their breath using the Elekta ABC system (Stockholm, Sweden). The IGTV was then created by incorporating a patient-specific interbreath-hold variation by combining all four sets of tumor contours from the four simulation CT sets (47). The clinical objectives were as follows: at least 95% of the PTV received 50 Gy and 100% of the IGTV received 50 Gy. The organs at risk (OAR) constraints were as the following: lung Dmean, V20 Gy and V10 Gy less than 4 Gy, 4.5% and 12%; esophagus max dose 34 Gy and V18.8 Gy less than 5 cc; heart max dose 34 Gy, V28 less than 15 cc; proximal chest wall V35 Gy and V30 Gy less than 1 cc and 30 ccs; trachea max dose 34.8 Gy and V15.6 Gy less than 4 cc; spinal cord max dose 26 Gy and V20.8 Gy and V14.5 Gy less than 0.35 cc and 1.2 cc. A RayStation treatment planning system (RaySearch Laboratories, Stockholm, Sweden) was used for plan and dose distribution calculations. Collapse Cone version 5.3 dose calculation was used for the dose distribution calculation.

### 2.5 Evaluation metrics

We evaluated the performance of deepPERFECT by using the root averaged squared sum of differences (RASSD) to compare the intensity of pCT scans with sCT scans, given as


Eq. 6 $$RASSD=\sqrt{\frac{1}{L*M*N}*\sum_{l=1}^{L}\sum_{m=1}^{M}\sum_{n=1}^{N}{\left(I_{pCT}\left(x_{l},y_{m},z_{n}\right)-I_{sCT}\left(x_{l},y_{m\;},z_{n}\right)\right)}^{2}}$$

where $x_{l}$ , $y_{m}$ , and $z_{n}$ are the Cartesian coordinates of the voxel. Secondly, the body, lung, and GTV contours on the two scans (Independently verified by the clinician) were compared using the Dice similarity coefficient (DSC)


Eq. 7 $$DSC=\frac{\left|X\cap Y\right|}{\left|X|+|Y\right|}$$

and Hausdorff distance (HD) sCT for the body and lungs.


Eq. 8 $$HD=\max\left(d_{H}\left(V_{p},V_{s}\right),d_{H}\left(V_{s},V_{p}\right)\right)$$

where $v_{p}$ , and $v_{s}$ are the coordinates of the contours on pCT and sCT.


Eq. 9 $$d_{H}\left(v_{p},v_{s}\right)=\max\left(\min\left(d\left(v_{p},v_{s}\right)\right)\right)$$

where d is the distance between two voxels. Because the lengths of the scans are different for sCT and pCT, as PET/CT and thus sCT include more body length, we only compared the bodies on overlapping Z-slices. Finally, for dosimetric comparison, we used point measurements of dose–volume histogram curves (DVH). We reported the median and range values for the initial plan (sCT plan), adaptation to position, and adaptation to shape scenarios.

## 3 Results

### 3.1 Evaluation of synthesized CT

**Figure 5:**
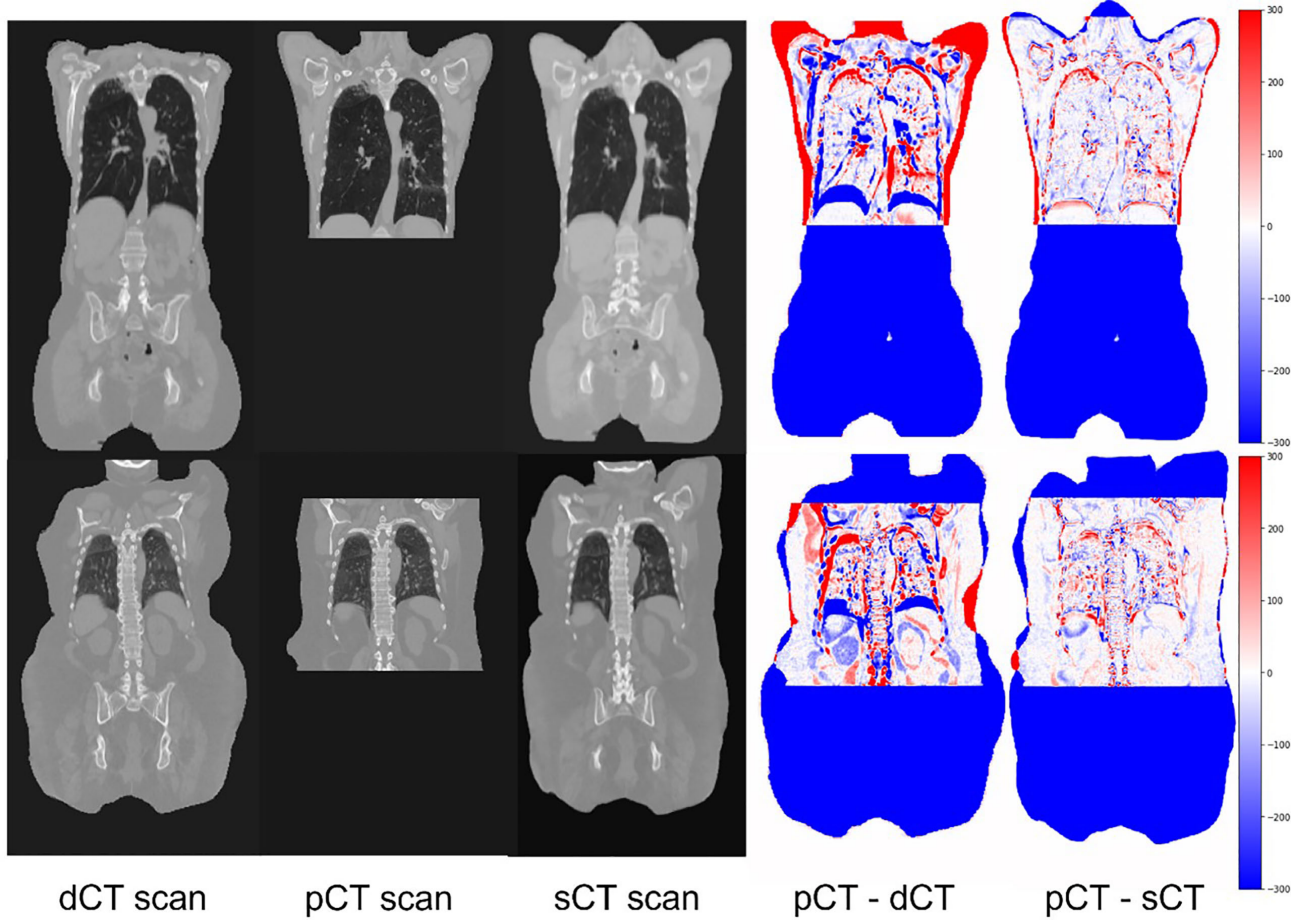
Example coronal slides extracted from two sample test cases. The input image, dCT (PET/CT scan), target scan (pCT), warped scan using the deepPERFECT generated DVFs (sCT), difference image pCT–dCT (target–input scan) (fourth column), and difference image pCT–sCT (target–output scan). The output scans high conformality with the target image for the overlapping area. Most prominently, the diaphragm level and lung bronchus, which are mainly affected by the ABC technique, are well aligned on sCT and target pCT scans.

**Figure 5** shows two sample cases for planning the CT synthesis. The scans were shown in a coronal view to emphasize diaphragmatic movement. The first three columns show the dCT, pCT, and sCT, respectively. The fourth and fifth columns show the differences between pCT, dCT, and sCT. As shown, deepPERFECT significantly reduced the difference between pCT and dCT, resulting in the same level of the diaphragm on the two scans.

We have summarized the quantitative evaluation of sCT compared with pCT using RASSD, DSC, and HD for the entire body and lungs in **Supplementary Table 1**. The difference between HU of the sCT and pCT was negligible and the body and lungs contours showed a high conformality reflected in average DSC of 0.91 and 0.97, and HD values of 7.9 and 4.9 mm for the body and lungs, respectively. As expected, due to day-to-day variation (77–82) and breathing motion, the GTV showed lower conformality. We also found 0% folding for the generated DVFs.

### 3.2 Evaluation of deformation vector fields

The curvature smoothness regularization curve (Eq. 5) enforces the smoothness of the DVFs and prevents folding; however, folding may still occur. Thus, we ensured the prevention of folding using diffeomorphic deformation. Mathematically folding occurred when the Jacobian determinant of the DVF matrix became negative. The Jacobian determinant is defined as


Eq. 10 $$\det\left(\text{J}\left(\text{x},\text{y},\text{z}\right)\right)=\det\left(\;{\left[\begin{array}{ccc} \frac{\partial }{\partial x} & \frac{\partial }{\partial y} & \frac{\partial }{\partial z} \end{array}\right]}^{T}*\;\left[\begin{array}{ccc} f_{x}\left(x,y,z\right) & f_{y}\left(x,y,z\right) & f_{z}\left(x,y,z\right) \end{array}\right]\right)$$

where $f_{x},\;f_{y}$ , and $f_{z}$ are the deformation vector fields in the x, y, and z directions, respectively. **Supplementary Figure 1** shows the overlay of the Jacobian determinant on the CT coronal view. As shown, the Jacobian determinants were primarily around 1 (yellow overlay), for some parts, they became close to zero (gold overlay) and no folding occurred (no red overlay).

### 3.3 Evaluation of radiotherapy plans

We evaluated the quality of the SBRT plans by comparing the DVH indices for the dose distributions of the initial sCT, ATP, and ATS plans. **Table 1** summarizes the median and range of DVH indices for all three dose distributions.

**Table 1:** Dosimetric comparison of the three planning scenarios.

DVH Index	sCT Plan			Adapt to Position			Adapt to Shape		
	Median	Min	Max	Median	Min	Max	Median	Min	Max
IGTV 50 Gy (%)	100	100	100	100	100	100	100	100	100
PTV 50 Gy (%)	95	95	95	94.1	92.3	96.2	95	95	95
Chest wall 45 Gy (cc)	0.18	0	0.57	0.14	0	1.34	0.23	0	0.95
Chest wall 30 Gy (cc)	3.14	1.99	4.9	3.6	0.39	5.95	3.89	1.02	5.24
Spinal Canal Max dose (cGy)	969	530	1,730	988	526	18,50	987	542	1,741
Spinal Canal 20 Gy (cc)	0	0	0	0	0	0	0	0	0
Lungs 5 Gy (%)	6.35	5.63	13.02	6.54	5	12.9	6.39	5.42	12.5
Lungs 10 Gy (%)	2.78	1.71	6.7	2.71	1.56	6.71	2.71	1.59	6.66
lungs 15 Gy (%)	1.52	0.98	5.01	1.47	0.88	4.99	1.51	0.94	4.99
lungs 20 Gy (%)	0.99	0.66	3.45	0.94	0.59	3.42	0.98	0.61	3.4
lungs 30 Gy (%)	0.52	0.31	1.59	0.48	0.29	1.58	0.51	0.31	1.57
Esophagus max (cGy)	959	331	1840	966	420	1,806	1,027	388	1,766
Esophagus 30 Gy (cc)	0	0	0	0	0	0	0	0	0
Heart max (cGy)	540x	0	897	504	0	1,040	631	0	1,045
Heart 40 Gy (cc)	0	0	0	0	0	0	0	0	0
Heart 20 Gy (cc)	0	0	0	0	0	0	0	0	0
Trachea 35 Gy (cc)	0	0	0	0	0	0	0	0	0
Trachea max (cGy)	0	0	841	0	0	815	0	0	812
Aorta max (cGy)	2,237	0	2,611	2,331	0	3,750	2,339	0	3,690
Aorta 30 Gy (cc)	0	0	0	0	0	0.21	0	0	0.29

For each plan, the initial sCT plan (left), adapted to position (middle), and adapted to shape (right), we show the median and range of clinically significant DVH indices. As shown, there is only a trivial target coverage loss with adaptation to the position plans which was rectified by adapting to the shape scenario.

Our results (**Table 1**) show that the target coverage can be preserved using our method. This was shown by the 100% coverage of the GTV with the prescribed dose for all scenarios and a negligible difference in PTV coverage. Moreover, we found comparable values for all OARs doses, with the chest wall showing the highest dose difference owing to the proximity of the GTV to the chest wall in some cases. Additional dosimetric evaluations are presented in **Supplementary Table 2**, which further shows that the preliminary plan can achieve comparable values for the spinal canal, aorta, and esophagus.

## 4 Discussion

This paper presented a deep learning platform for the adaptation of a diagnostic PET/CT scan (dCT) to planning CT (pCT) in NSCLC. It can compensate for the changes in the patient body shape and image acquisition setup between the two scans. Although dCT has been used for RT planning (22–24), we demonstrated in **Figure 2** that the anatomical changes between dCT and pCT lead to different dose distributions; thus, dCT cannot be used directly for treatment planning, while we are capturing these differences with our novel deepPERFECT imaging method. This method is superior to using dCT planning for initial planning, as it incorporates the changes in the shape of the patient’s body and diaphragm level in planning to provide a more realistic dose distribution. Thus, with deepPERFECT, we consider prior knowledge of the patient body and treatment setup. Therefore, deepPERFECT allows the implementation of the DAART approach.

To train the model, we employ the multiterm cost function shown in Eq. 1. We found that curvature smoothing regularity is essential for the generation of plausible deformation vector fields. Moreover, it facilitated the training process of the model. In addition, we used a novel contrast-fidelity term (Eqs. 3 and 4). One of the major considerations in lung CT is the details of the lung region. In practice, physicians enhance the visualization of these details using a specific level and window that are otherwise barely visible on the original CT. In deepPERFECT, we trained the model using our novel contrast fidelity term so that it could see the lung CT as the physician sees. Be that as it may, the use of image similarity loss (Eq. 2). It is still imperative to balance the lung features and body shape.

**Supplementary Table 1** shows the results of the quantitative assessment of sCT and contours. Our results confirm previous studies that dCT is suitable for planning (22, 24, 83), and sCT has comparable HU for the body, lungs, and GTV structures. The deepPERFECT-generated body and lung shapes showed a high conformality to the patient’s body on the pCT scan. Our unique approach to applying diffeomorphic vector fields ensures plausible and realistic contours by preventing folding and tearing. We confirmed the plausibility of the contours by calculating the Jacobian determinant (**Supplementary Figure 1**); thus, we believe that our approach is a robust DVF generation method for medical image adaptation.

Although the body and lungs showed high conformality (**Supplementary Table 1**), the GTV had the lowest spatial overlap between the sCT and pCT. This was expected mainly because of the small size of the GTV compared to the entire body (median of 3.1 cc), and DSC had a positive correlation with GTV size. As a result, by simply copying the sCT plan and recalculating the dose on the pCT scan, we achieved low PTV coverage. This can be mitigated by plan adaptation using the proposed expeditious adaptive workflow.

DeepPERFECT is an invaluable tool for expeditious image-guided RT, as it is highly compatible with recent advanced adaptive RT (ART) systems. Traditional patient CT sim can be avoided, and the ART system can image and adapt the preliminary plan generated from synthetic CT to the current patient anatomy on treatment day1. Currently, several ART systems are available such as MRIdian (ViewRay, Oakwood Village, Ohio, USA), UNITY MR-linac (Elekta AB, Stockholm, Sweden), and Varian Ethos (Varian Medical Systems, Palo Alto, CA). For instance, the UNITY system provides two different workflows for plan adaptation: adapting to position (ATP) and adapting to shape (ATS) (84, 85). Here, we showed that the deepPERFECT-enabled DAART (**Figure 3**) resulted in good plan quality for the adaptation scenarios (**Table 1**). ATS adaptation of the early plan (sCT plan) to pCT resulted in a good plan for treatment delivery. More importantly, the power of deepPERFECT is more prominent in achieving a non-inferior plan by just ATP, which resulted in minimal PTV coverage loss (less than 3%) in four out of five cases.

One concern may be that all the work is happening in the adaptation stage rather than the synthetic CT and preliminary RT plan stage, but the use of simulation/planning-CT-free ART is limited to disease sites with limited anatomical variation, such as the brain, head, and neck. In contrast, the anatomy of patients with lung cancer can vary greatly, as shown in **Figure 2**. The difference between diagnostic PET-CT and planning CT can result in significant changes in the relative spatial locations of tumors and OARs, which may lead to dosimetric inaccuracies and suboptimal dose constraints. For instance, RT-planning CT may have a larger lung volume, and these constraints may restrain plan optimization, resulting in suboptimal tumor coverage. Significant anatomical differences require radiation oncologists to re-evaluate the spatial relationships between targets and OARs and make trade-off decisions between tumor coverage and OAR sparing, as well as their dose constraints. The requirement for additional evaluation and decision-making immediately before RT delivery owing to unexpected anatomical differences can further increase the time and complexity of ART, which is a time-consuming process. It is important to mention that the purpose of IGRT and online geometric adaptive strategies is to react to changes in patient configuration under the same setup guidance. The combination of the method developed here, and ART can extend the capabilities of ART to scenarios where there is a large difference in patient configuration between diagnosis and RT treatment.

Our study had a few limitations. First, owing to GPU limitations, we had to down-sample the scans to 2.5 mm voxel size so that the network field of view (128 cubic patch) encompasses a decent view of the body. Our initial experiments showed that the network showed the best performance when it had a large view of the body rather than small patches. Future studies will use the original image rather than a downsampled scan. Another limitation is the small amount of data, which was limited to one institution used in this study. To overcome this limitation, we performed a rigorous evaluation of our method using several image-similarity metrics and RT-planning dosimetric indices. More importantly, our virtual clinical trial demonstrated the practicality of this method and future studies are aimed at clinical trials with larger cohorts.

In future studies, we will investigate possible solutions for using full-resolution CT scans with no down-sampling, conduct multi-institutional clinical trials, and extend the application of deepPERFECT to other anatomical sites. Here, we showed the feasibility of deepPERFECT for lung cancer and to enhance the performance of the system; a larger cohort will be used for future studies.

## 5 Conclusion

This paper presents a novel deep accelerated adaptive RT (DAART) approach to address the challenge of the current considerably lengthy RT workflow and implement an expeditious RT treatment course. We demonstrated the effectiveness of DAART for NSCLC radiotherapy in a virtual clinical trial. Our method features a novel deepPERFECT system that adapts and transforms the patient diagnostic scan to a treatment initiation setup that allows the initial RT treatment plan for the early assessment and evaluation of RT plans and deciding on dose trade-offs. We introduced a novel contrast fidelity term that significantly enhanced the performance of DL models to capture the relevant details of medical images for different applications. Using deepPERFECT, we achieved state-of-the-art CT transformation accuracy and an expedited framework for adaptive radiation therapy with at least 50% shorter wait time.

## Data availability statement

The raw data supporting the conclusions of this article will be made available by the authors, upon request without undue reservation.

## Ethics statement

The studies involving human participants were reviewed and approved by JHU IRB. Written informed consent for participation was not required for this study in accordance with the national legislation and the institutional requirements.

## Author contributions

The study was designed by all authors. HH and KD prepared the manuscript and HH, QC, XF, RZ, RF, KRV, RKH, YD, XJ, and KD contributed to data analysis and interpretation. HH and KD participated in collecting data. All authors contributed to the article and approved the submitted version.
