# Supplementary material for: DAART: a deep learning platform for deeply accelerated adaptive radiation therapy for lung cancer
Source: Front Oncol. 2023 Jul 6;13:1201679. doi: 10.3389/fonc.2023.1201679 (PMC10359160; doi:10.3389/fonc.2023.1201679)
Supplement: Supplementary file 1 [file DataSheet_1.docx]

Supplementary Material

DAART: a Deep Learning Platform for Deeply Accelerated Adaptive Radiation Therapy for Lung Cancer

**Hamed Hooshangnejad^1,2,3^, Quan Chen^4^, Xue Feng^5^, Rui Zhang^6^, Reza Farjam^2^, Khinh Ranh Voong^2^, Russell K. Hales^2^, Yong Du^7^, Xun Jia^2^, Kai Ding^2,3,*^**

^1^Department of Biomedical Engineering, Johns Hopkins School of Medicine, Baltimore, MD, USA

^2^Department of Radiation Oncology and Molecular Radiation Sciences, Johns Hopkins School of Medicine, Baltimore, MD, USA

^3^Carnegie Center of Surgical Innovation, Johns Hopkins School of Medicine, Baltimore, MD, USA

^4^Department of Radiation Oncology, City of Hope Comprehensive Cancer Center, Duarte, CA, USA

^5^Carina Medical, Lexington, KY, USA

^6^Division of Computational Health Sciences, Department of Surgery, University of Minnesota, Minneapolis, MN, USA

^7^Department of Radiology and Radiological Science, Johns Hopkins School of Medicine, Baltimore, MD, USA

*** Correspondence:**Corresponding Author
kding1@jhmi.edu

Supplementary Table 1. The result of the image quality evaluation

| Metric | Mean | (Min, Max) |
| --- | --- | --- |
| Body RASSD (HU) | 83 | (33, 108) |
| Body DSC | 0.91 | (0.89, 0.94) |
| Body HD (mm) | 7.9 | (6.8, 9.1) |
| Body Folding (%) | 0.0 | (0.0,0.0) |
| Lungs RASSD (HU) | 14 | (3, 21) |
| Lungs DSC | 0.97 | (0.96,0.98) |
| Lungs HD (mm) | 4.9 | (3.3, 5.8) |
| GTV RASSD (HU) | 73 | (12, 134) |
| GTV DSC | 0.64 | (0.51, 0.89) |
| GTV HD (mm) | 4.1 | (2.2, 5.1) |


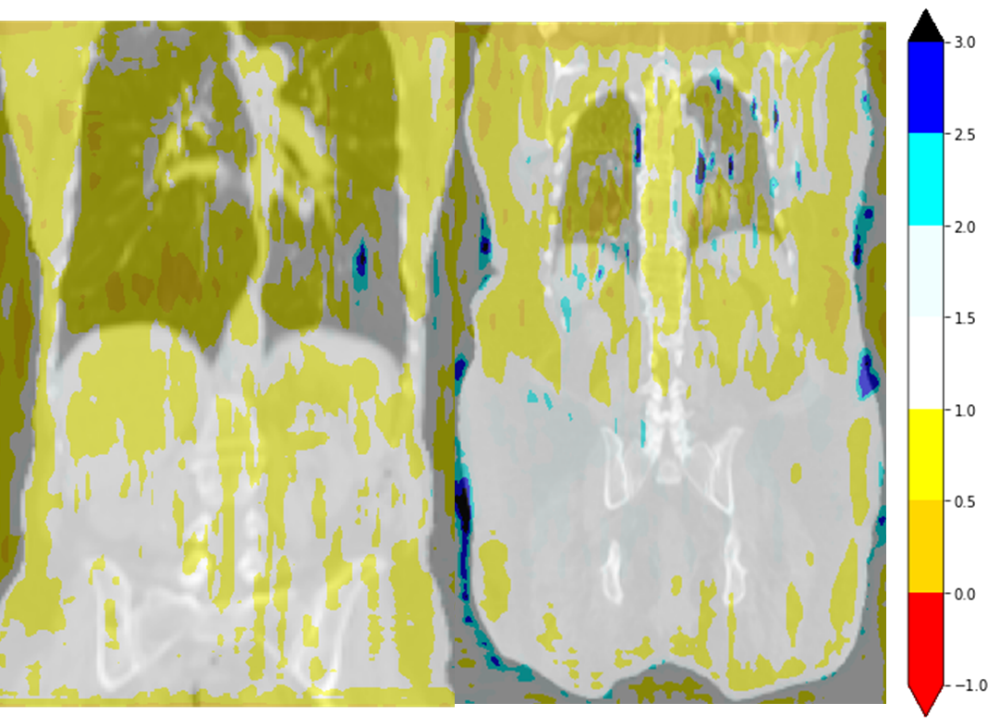


Supplementary Figure 1. Example of Jacobian colormaps overlaid on the coronal slide of dCT scan for two sample test cases. The volume changes are smooth, shown by the Jacobian determinant of mostly around 1 (yellow overlay). Zero percent folding was found for generated DVFs.

Supplementary Table 2. Additional Dose Volume Histogram indices

| DVH Indices | sCT plan | | Adapt to Position | | Adapt to Shape | |
| --- | --- | --- | --- | --- | --- | --- |
|  | Median | (Min, Max) | Median | (Min, Max) | Median | (Min, Max) |
| Esophagus V34Gy (cc) | 0 | (0, 0) | 0 | (0, 0) | 0 | (0, 0) |
| Esophagus V18.8Gy (cc) | 0 | (0, 0) | 0 | (0, 0) | 0 | (0, 0) |
| Aorta V49Gy (cc) | 0 | (0, 0) | 0 | (0, 0) | 0 | (0, 0) |
| Aorta V43Gy (cc) | 0 | (0, 0) | 0 | (0, 0) | 0 | (0, 0) |
| Spinal Cord V26Gy(cc) | 0 | (0, 0) | 0 | (0, 0) | 0 | (0, 0) |
| Spinal Cord V20.8Gy (cc) | 0 | (0, 0) | 0 | (0, 0) | 0 | (0, 0) |
| Spinal Cord V14.8Gy (cc) | 0 | (0, 0.17) | 0 | (0, 0.26) | 0 | (0, 0.18) |
| Spinal Cord Dmax (cGy) | 969 | (530, 1730) | 988 | (526, 1801) | 981 | (560, 1741) |
